# The fault in his seeds: Lost notes to the case of bias in Samuel George Morton’s cranial race science

**DOI:** 10.1371/journal.pbio.2007008

**Published:** 2018-10-04

**Authors:** Paul Wolff Mitchell

**Affiliations:** Department of Anthropology, University of Pennsylvania, Philadelphia, Pennsylvania, United States of America

## Abstract

The discovery of nearly 180-year-old cranial measurements in the archives of 19th century American physician and naturalist Samuel George Morton can address a lingering debate, begun in the late 20th century by paleontologist and historian of science Stephen Jay Gould, about the unconscious bias alleged in Morton’s comparative data of brain size in human racial groups. Analysis of Morton’s lost data and the records of his studies does not support Gould’s arguments about Morton’s biased data collection. However, historical contextualization of Morton with his scientific peers, especially German anatomist Friedrich Tiedemann, suggests that, while Morton’s data may have been unbiased, his cranial race science was not. Tiedemann and Morton independently produced similar data about human brain size in different racial groups but analyzed and interpreted their nearly equivalent results in dramatically different ways: Tiedemann using them to argue for equality and the abolition of slavery, and Morton using them to entrench racial divisions and hierarchy. These differences draw attention to the epistemic limitations of data and the pervasive role of bias within the broader historical, social, and cultural context of science.

## America’s founding race scientist: Biased science or biased scientist?

Samuel George Morton (1799–1851, Fig 1) and his collection of hundreds of human skulls are foundational to the history of scientific racism. Morton, a Philadelphia physician and naturalist, collected and compared crania from across the globe [1,2]. His comparative measurements of “internal capacity” (IC), the volume of the brain case, a proxy measure of brain size, were used to rank the relative intelligence of human races, producing a scientific justification for white, Anglo-Saxon superiority [3,4], manifest destiny [5], and the enslavement of Africans [6,7].

**Fig 1:**
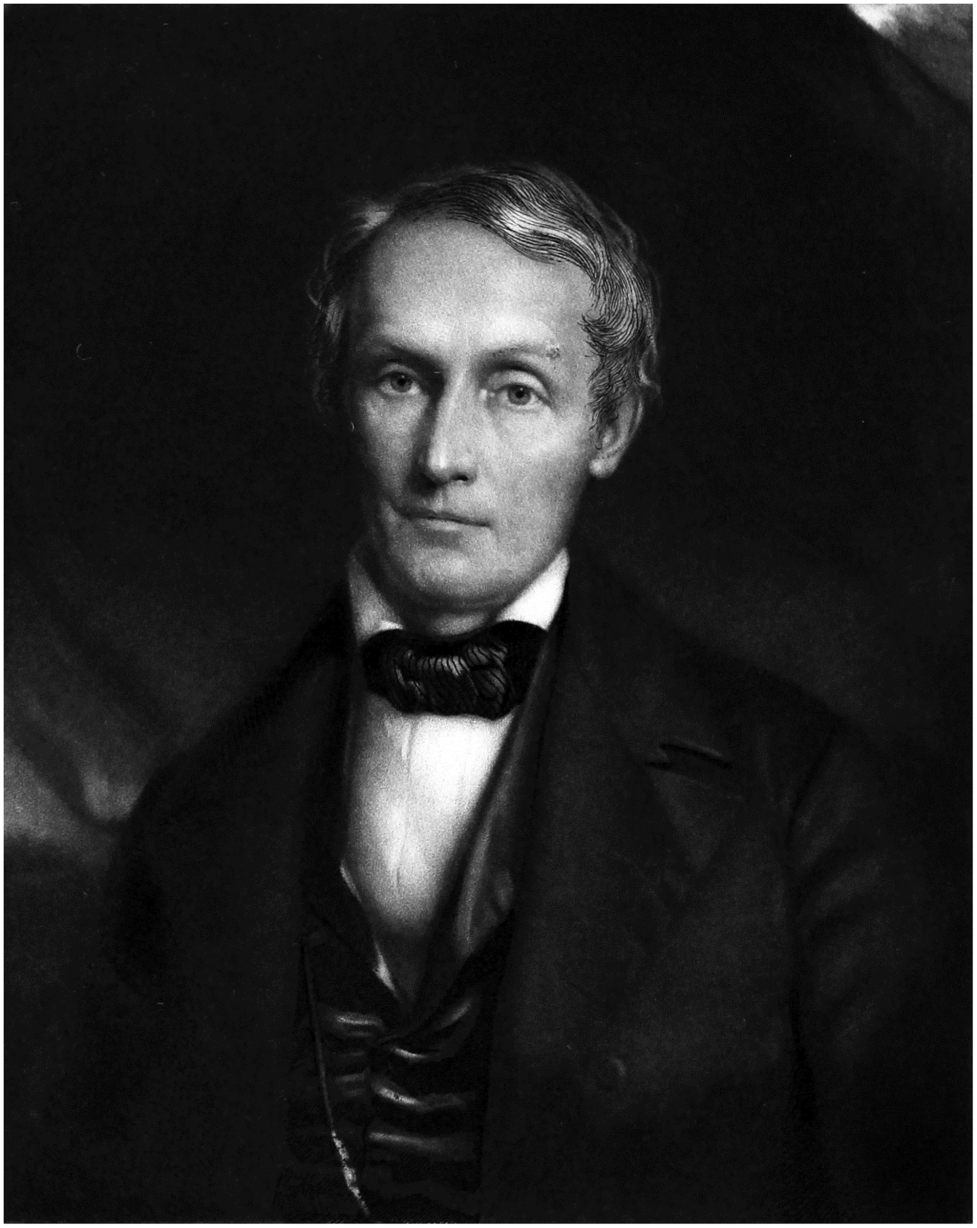
Samuel George Morton (1799–1851). Image available from: https://commons.wikimedia.org/wiki/File:Samuel_George_Morton_portrait.jpg.

The methods of Morton’s internationally recognized [8] cranial race science found legitimization in two occasionally intertwined intellectual currents. One flowed from systematic attempts at human racial classification on the basis of comparative anatomy, which began in Europe around the mid- to late 18th century [9,10,11]. The other arose from widespread interest in correspondences among cranial form, the brain, thought, and behavior, exemplified not only in the writings of influential anatomists such as Georges Cuvier [12] but also by the popularity of phrenology in the early to mid-19th century [13,14,15,16]. The assumptions necessary to consider the measurement of racial differences in brain size as a valid and sufficient explanation of racial differences in behavior and intelligence were shared among many mid-19th century natural historians and anatomists [17]. Moreover, such measurements were important in providing empirical fodder for longstanding debates about the unitary or separate origins of human races, respectively known as monogenism and polygenism [4,6,18,19], with obvious consequences for contemporary political questions regarding race-based slavery and colonialism [1,4,7,20]. In assembling and measuring the skulls of his “American Golgotha,” only possible because of his central position in Philadelphia’s budding Academy of Natural Sciences [2,21], Morton presented a quantitative argument not only for racial hierarchies of intelligence, but also for the separate origins of the races, elevating racial differences among humans to differences among species. In his final years, Morton’s attention focused on the deleterious effects of racial mixing, which he likened to hybridity among animals [22,23,24,25,26,27,28,29].

Once hailed a leading American scientist and recognized as the first physical anthropologist in the United States [30,31,32,33,34,35], Morton’s star fell through the late 19th century as evolutionary thought outmoded his assumptions of divinely fashioned racial divisions [18,19]. Morton was largely obscure by 1978, when American paleontologist and historian of science Stephen Jay Gould (1941–2002) claimed in *Science* that Morton’s cranial measurements were riddled with unconscious racial bias [36]. This charge was reprised in 1981 in an opening chapter of the bestselling *Mismeasure of Man* [37,38]. While criticism of Morton’s measures, methods, and racial categories had been published since the mid-19th century [24,39,40,41,42,43,44,45], the heat of late 20th century debates about racial bias in intelligence testing [46,47] and critical discussion of “biological determinism” provoked by sociobiology [48,49,50] generated ample interest in Gould’s exposé of Morton as a sterling case of scientific bias.

**Fig 2:**
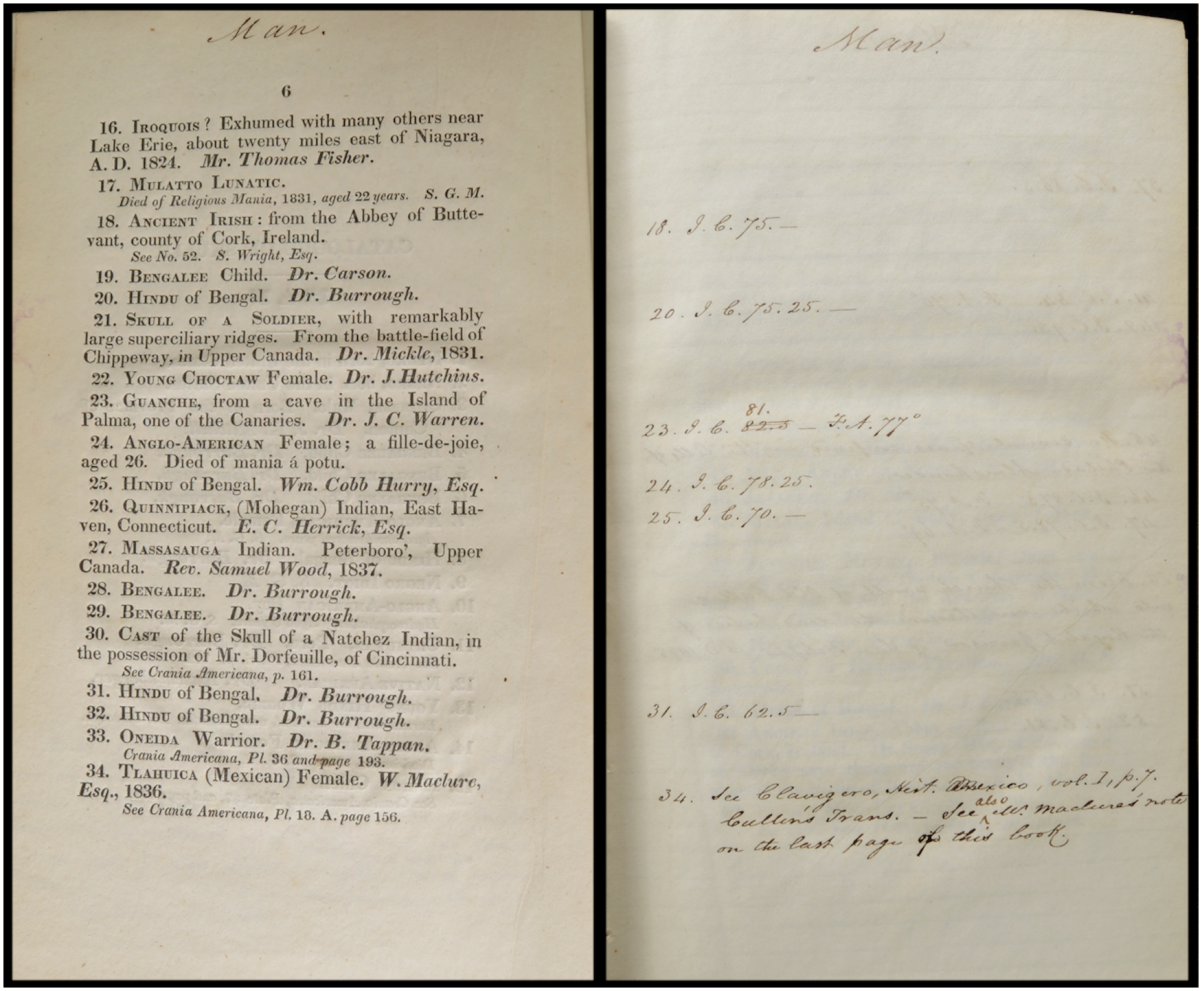
Excerpt from Morton’s personal copy of his *Catalogue of Skulls* (1840). These two exemplar pages show Morton’s handwritten notes on the right compared to the printed material on the left. Note that the handwritten IC data here are different than later-published shot IC data for the same crania (S1 Data part 1 and part 2). Image available from: https://archive.org/details/catalogueskulls00mort. IC, internal capacity.

Gould’s procedure was to analyze Morton’s published data and uncover “inconsistencies and shifting criteria,” “procedural omissions,” “slips,” “miscalculations,” and other errors, many of which tended toward Morton’s alleged a priori racial bias [36]. However, in 1988, Michael [51] re-measured many of Morton’s crania (which have been curated at the Penn Museum in Philadelphia since 1966), concluding the overall accuracy of Morton’s data. In 2011, Lewis and colleagues [52], with a more refined methodology, also re-measured and definitively showed the accuracy of Morton’s measures while also indicating some of Gould’s own analytical errors. However, as well as garnering significant media attention [53,54,55], Lewis and colleagues attracted criticism [56,57,58] for neither addressing nor defeating one of Gould’s central arguments about Morton’s measures. Presented here are handwritten notes inscribed by Morton in his personal copy of the first edition (1840) of his *Catalogue of Skulls* (Fig 2), archived at the Academy of Natural Sciences of Drexel University and now available for online, open-access viewing [59]. These handwritten notes, heretofore unacknowledged in print, have revealed new data to directly address Gould’s major outstanding argument against Morton and reframe a lingering debate about Morton and bias in scientific racism.

## Morton’s measures and Gould’s argument

**Fig 3:**
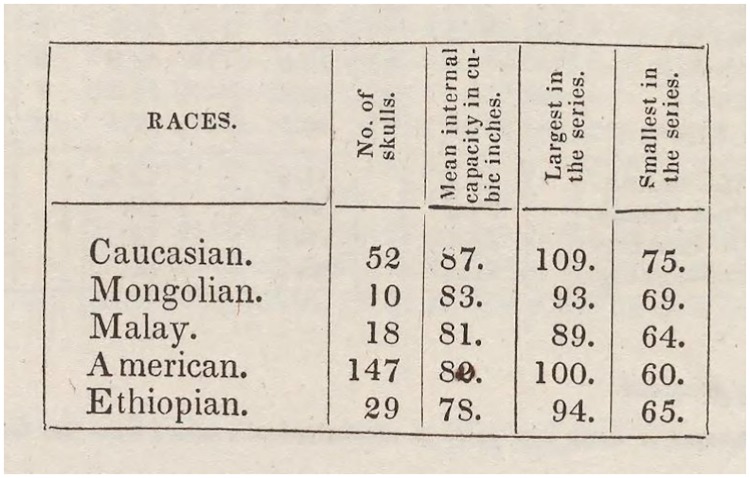
Morton’s cranial capacity by race in *Crania Americana* (1839) [60], page 260. Note Morton’s handwritten correction of the American mean in printed copies of *Crania Americana*, which he changes to 80 in^3^ from the incorrectly printed 82 in^3^. Image available from: https://archive.org/details/Craniaamericana00Mort.

Morton published three major works documenting his cranial collection: *Crania Americana* (1839) [60], *Crania Aegyptiaca* (1844) [61], and a *Catalogue of Skulls of Man and the Inferior Animals*, in three editions (1840, 1844, and 1849) [59,62,63]. Each successive edition of the *Catalogue* documented every skull in Morton’s growing collection at the time of publication. In *Crania Americana*, Morton published IC measures for the five principal races he recognized (Ethiopian [i.e., African], [Native] American, Caucasian, Malay, and Mongolian, Fig 3, S1 Text), taken by pouring white pepper seed into the brain cavity, then measuring the volume of seed in cubic inches necessary to fill the skull [60]. (Later, Morton [63] claimed that he had used “white mustard seed.” In recent years, white peppercorns, *Piper nigrum*, have been found still lodged in skulls in Morton’s collection, suggesting that peppercorns were used for measurement.) In *Crania Aegpytiaca* and the last edition of the *Catalogue of Skulls*, Morton published new IC data taken with lead shot because he had found inconsistencies in measuring IC with seed [63]. While Lewis and colleagues and Michael showed the accuracy of Morton’s lead shot measurements, Gould assumed the accuracy of the shot ICs [58]. (“I will assume, as Morton contends, that measurements with shot were objective and invariably repeatable to within 1 in^3^” [36].) The bias that Gould attributed to Morton was to be found in the difference between seed and shot ICs for racial groups. Gould contended that seed, but not shot, could easily be manipulated as Morton packed and settled the seeds into the cranial cavity in the measurement of each skull. Morton, Gould supposed, had likely unconsciously overstuffed Caucasian crania with seed and only sparingly filled the crania of other races, leading to a systematic underestimation of non-Caucasian IC with seed.

Demonstrating this supposed bias is not straightforward, however, because *Crania Americana* records IC (seed) only as an average, minimum, and maximum for all races but one, the American, for which the full data for all 147 crania measured are published. In contrast, the 1849 *Catalogue* lists the IC (shot) of every cranium measured, for all races. Thus, direct comparisons of seed and shot ICs from Morton’s published works are possible only for the American crania. Of the 147 American crania measured in *Crania Americana*, 111 were later re-measured with shot and recorded in Morton’s 1849 *Catalogue* (See Supporting Information Data 3: Native Am Seed Shot, from [52]). These 111 comparisons show a mean difference between seed and shot (“seed-to-shot correction”) of +2.2 in^3^ for Americans. Inferring the mean seed-to-shot correction for the other four races requires reconstructing which crania were measured in 1839, which Gould did by working from the information recorded in the third edition of the *Catalogue* (1849) about the timing and ordering of Morton’s acquisition of crania. Thereby, he could presumably account for the crania in Morton’s collection by the time of *Crania Americana*’s publication. Comparing the seed IC means for Africans and Caucasians published in 1839 to the shot IC means of Gould’s reconstructions of which crania were re-measured in each racial group, Gould produced a mean seed-to-shot correction of +5.4 in^3^ for Africans and +1.8 in^3^ for Caucasians (Gould did not analyze the Mongolian or Malay seed-to-shot corrections). Gould suggested that such a difference between Africans and Caucasian corrections is most likely explained by Morton’s underestimation of African crania with seed, prima facie evidence of Morton’s bias [36]. Gould suggested that this bias was unconscious because Morton, commendably, openly published his data [36] (as this paper shows, contra Gould, Morton published many—but not all—of his data).

Although he proffered unconscious bias as an explanation, Gould also mentioned what now appears the likelier source of the seed-to-shot correction differences: Morton “borrowed some skulls from friends” in 1839 [36]. Gould, although probably not entirely accurately (S2 Text), identifies only 18/29 (or 62%) of the African crania and 19/49 (or 39%) of the Caucasian crania as having been re-measured, while the rest were borrowed from Morton’s associates in 1839 and not later re-measured. Thus, in calculating his “seed-to-shot corrections,” Gould compares the shot IC means of the 18 re-measured Africans and 19 re-measured Caucasians to the seed IC means for 29 Africans and 49 Caucasians measured in *Crania Americana*. In so doing, he does not account for the other 11 Africans and 30 Caucasians measured in *Crania Americana*, which comprise approximately 40% to 60% of their respective samples, as has been briefly noted before [52]. Given that borrowed skulls that were not re-measured comprise such a significant proportion of Morton’s 1839 samples, it is likely sample differences, rather than systematic bias in seed measurements, that accounts for the differences in seed and shot IC (S3 Text). Moreover, an enlarged sample of direct seed—shot comparisons possible with the recovered seed data presented here does not support Gould’s claim of Morton’s unconscious bias.

## The seed data

Previously, no seed IC data for non-American crania were known. However, Morton’s handwritten notes in his personal, signed-and-dated copy of the printed 1840 *Catalogue* contain seed measures for at least 51 crania, including, using Morton’s racial groupings, 5 Africans, 24 Caucasians, 2 Mongolians, 14 Malay, 4 “Ancient Caucasians” (Ancient Egyptians), and 2 “mixed race” persons (Table 1; S1 Data part 1). These IC were inscribed with ink in Morton’s handwriting in his *Catalogue*, next to the entry for the corresponding cranium. Three observations strongly suggest that these handwritten data are some of the “missing” seed data [56]. First, Morton notes that he had begun to re-measure crania with shot in the spring of 1841 [64], suggesting that any measures recorded around 1840, when the *Catalogue* was printed and dated with Morton’s signature, were of seed. Second, these handwritten ICs differ, often significantly, from the later-published shot IC [61,63] for each corresponding cranium (S4 Text). Third, Morton’s handwritten ICs include crania from Scottish phrenologist George Combe’s (1788–1858) collection. Combe wrote the “Phrenological Appendix” to *Crania Americana* and traveled with a cranial collection while giving lectures on phrenology across the US between 1838 and 1840 [65]. Morton’s handwritten IC notes in the 1840 *Catalogue* include IC measures of a “Lowland Scot” and “Swiss” noted specifically as belonging to Combe [59]. Both of these crania are measured in *Crania Americana* [60] and were not later accessioned into Morton’s cranial collection. Thus, the handwritten IC for these skulls must be seed because they were recorded around the time of *Crania Americana*’s composition. Plausibly, Morton recorded these measures in 1839 and then hand-copied them into his 1840 *Catalogue*. The handwritten ICs in the 1840 *Catalogue* do not include the ICs of American crania, presumably because these were published in *Crania Americana*, further suggesting that these seed measures were recopied after having been initially recorded elsewhere.

**Table 1:** Differences between seed and shot IC race groups follow Morton (1849) (S1 Text). See S1 Data (part 1) for full table of Morton’s (1840) handwritten IC American data calculated from difference between 1839 and 1849 published IC (see S1 Data [part 3]).

	Mean	SD	Min	Max	Av. Percent	Number	Number *C*. *Am*.
**American**	2.2	2.8	−10	12	2.9	111	147
**Caucasian**	3.4	1.4	0.5	6.5	4.3	22	52
**Ethiopian**	1.9	1.7	−0.5	4	2.3	5	29
**Malay**	4.3	2.9	1.5	13	5.5	13	18
**Mongolian**	2	0	2	2	2.5	2	10

**Abbreviations**: Av. Percent, average percentage difference between Morton (1840) handwritten IC and Morton (1849) IC; Number, number of handwritten ICs in Morton (1840) for this race group; Number *C*. *Am*., number of sample size for this race group in *Crania Americana*, IC, internal capacity.

**Fig 4:**
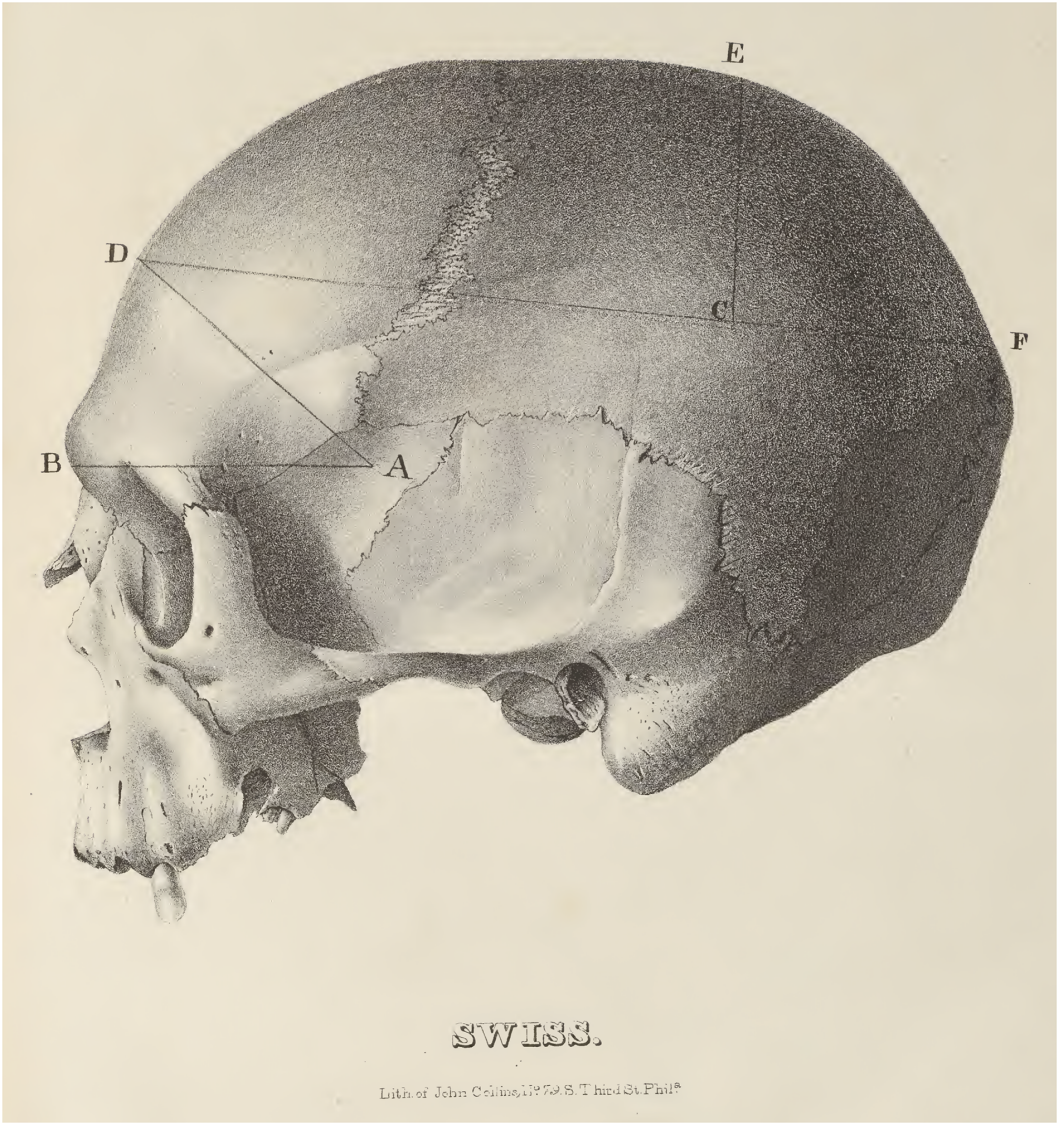
Lithograph of Swiss skull, plate 71 of *Crania Americana* [60]. This skull was among dozens that Morton borrowed from colleagues, measured, and sometimes illustrated for inclusion in *Crania Americana*, accounting for the sample differences in “racial groups” between this work [60] and Morton’s later [62,63] craniological publications. Image available from: https://archive.org/details/Craniaamericana00Mort.

Morton’s handwritten ICs do not account for the full sample for the IC mean of any racial group in *Crania Americana* (see difference between “Number” and “Number *C*. *Am*.” in Table 1). It is presently impossible to determine how much of this discrepancy is due to Morton not having recorded seed IC measurements for crania in his possession in his 1840 *Catalogue* or to Morton having borrowed crania for his 1839 sample, but both factors contributed. Regarding the first factor, Morton certainly did not hand-copy every seed IC measurement into his 1840 *Catalogue*. For example, Morton writes in *Crania Americana* [60] that some of the African crania measured for the African (“Ethiopian”) sample were sent from Dr. Robert McDowell in Liberia. At least four crania, numbers 645–648, were sent by McDowell and their entries printed in the 1840 *Catalogue*, but Morton does not record their seed ICs. (Red wax stains on the pages of his 1840 *Catalogue* show where Morton had affixed small, loose paper notes. These are now missing. The outstanding ICs may have been written on these.) Regarding the second factor, it is also evident that Morton measured multiple borrowed crania with seed. In 1839, Morton published measures not only of the Scot and Swiss skull but also four “Esquimaux” crania from Combe’s collection [60,66]. (Curiously, Gould repeatedly chose illustrations of the skulls that Morton had borrowed as iconic of Morton’s collection. A lithograph of Combe’s Swiss skull, mentioned above, is plate 71 in *Crania Americana* and is featured on the cover of Gould’s 1981 edition of the *Mismeasure of Man* [Fig 4]. Additionally, Gould included the lithograph of these four “Esquimaux” crania, plate 70 in *Crania Americana*, as the only image of the skulls in his 1978 *Science* article [36].) Combe was not the only source of borrowed crania. Although it is not attributed to anyone in the printed edition, Morton’s handwritten notes in his 1840 *Catalogue* also indicate that he acquired a cranium (number 98) from fellow Philadelphia physician Thomas Dent Mutter (1811–1859). Physicians like Mutter, as well as the College of Physicians of Philadelphia [67] and Philadelphia’s Central Phrenological Society, America’s oldest phrenological society [68], certainly had other crania that would have been convenient for Morton to borrow and measure, and likely account for many of the remaining crania from the 1839 sample.

The data that have been recovered do allow for direct comparison of individual seed and shot measures for 42 crania beyond the 111 American crania for which Morton published both seed and shot measures (S1 Data part 2). Single factor one-way ANOVA of these seed—shot differences for the five categories analyzed in 1839 (S1 Data part 3) suggests that there is no statistically significant (at *p* < 0.05) difference among the means of corrections for any group, except for the Malay group. When one +13 in^3^ outlier (number 572) is removed, the significance for the Malay disappears. Because between about 80% and 28% of the seed data for each racial group are still missing, Gould’s claim of Morton’s bias in seed measures may yet stand, but there is no evidence for it in the only relevant seed data known—presented here—and its plausibility is significantly hampered by the sample differences between 1839 and 1849, noted above.

By 1841 [64], Morton was aware of the errors in the seed measurements, which he explained were made by both him and his assistant [63]. (Morton’s measuring assistant was likely J. S. Phillips [1800–1876], as indicated by the text in the dedication to Phillips in *Crania Americana*. Whatever bias may exist in the seed measurements must be attributed to Phillips also, not only Morton [11].) The current data underscore the claim that errors in the seed measurements were significant, but likely random (S5 Text). Upon realizing the errors in the seed measurements taken with his assistant, Morton began taking all measurements in shot and by himself. On the cover of his personal 1840 *Catalogue*, Morton penciled 20 additional ICs, which match later published shot measures for the same crania. These are apparently among the first shot measurements that Morton recorded (S4 Text).

## Bias: More than just results

Gould had more criticisms of Morton than the difference between seed and shot measurements. These have already been discussed and debated in detail elsewhere [11,35,51,52,56,57]. Many of Gould’s criticisms, and more, can be found among 19th century commentaries on Morton’s work [24,39,40,41,42,43,44,45]. However, as suggested by Weisberg and Paul [58], “the measurement issue” remains Gould’s novel, outstanding, and perhaps strongest argument for Morton’s unconscious bias in his cranial race science. While this analysis of the new seed data does not support Gould’s claim of Morton’s unconscious bias as revealed in his seed measures, Morton’s results cannot be said to be free of significant impact by his racial biases [52]: Gould’s general diagnosis of Morton’s “a priori conviction of racial ranking so powerful that it directed his tabulations along preestablished lines” [36] remains perceptive.

The nature of Morton’s biases is not hard to discern. In the mass of traveler’s reports, ethnological and historical writings, and observations of foreign peoples that he compiled and commented upon in *Crania Americana*, *Crania Aegyptiaca*, and his other works, although he noted diversity in mental capacities within each “race” and judged some as evidently mentally superior and more “susceptible to cultivation” than others, Morton characterizes many non-Caucasians in often repugnant terms [60]. Unsurprisingly, Morton’s estimation of the mental capacities of the races rather neatly mirrors the hierarchy of cranial sizes he reports.

Even among Caucasians, Morton saw hierarchy. Possessing the prejudices typical of 19th century American racial Anglo-Saxonism, Morton held Caucasians as superior to other races, and Teutonic (Germanic) peoples such as the English as superior to other Caucasians [3]. (In 1849, he specifies the “Teutonic Family” as having the largest cranial capacity of all human groups [63].) On page 17 of *Crania Americana* [60], he describes “that extraordinary people whom we call the English or Anglo-Saxons” as follows: “Inferior to no one of the Caucasian families in intellectual endowments, and possessed of indomitable courage and unbounded enterprise, it has spread its colonies widely over Asia, Africa and America; and, the mother of the Anglo-American family, it has already peopled the new world with a race in no respect inferior to the parent stock.” In contrast, Morton, whose father descended from an English colonist family [69,70] in Clonmel, southern Ireland, opined without citation on page 16 [60] that “the most unsophisticated Celts are those of the southwest of Ireland, whose wild look and manner, mud cabins and funereal howlings, recall the memory of a barbarous age.”

Comparing Morton to contemporaneous anthropologists and craniologists, such as Johann Friedrich Blumenbach (1752–1840) [71,72,73], James Cowles Prichard (1786–1848) [74,75], and Friedrich Tiedemann (1781–1861, Fig 5) [76,77,78,79], who did not share Morton’s polygenist, hierarchical, and static conception of racial difference, renders apparent the influence of these views on Morton’s work. Although Morton cites Blumenbach and Prichard repeatedly, comparison with Tiedemann—whose craniological research Morton only mentions, curiously, in posthumously published papers [28]—is most instructive. German anatomist and physiologist Friedrich Tiedemann’s novel investigation of racial differences in cranial capacity, published in 1836 in the *Philosophical Transactions of the Royal Society* [76], was the first study of its kind. After measuring 248 crania from five racial groups, Tiedemann concluded that the wide and overlapping ranges of cranial capacity in each race suggested no racial differences in cranial capacity and intelligence: “The principal result of my researches on the brain of the Negro, is, that neither anatomy nor physiology can justify our placing them beneath the Europeans in a moral or intellectual point of view” [76]. Tiedemann expanded this study with the measurement of over 200 additional crania and published it in German in 1837 with the same conclusion of equality for all races [77].

**Fig 5:**
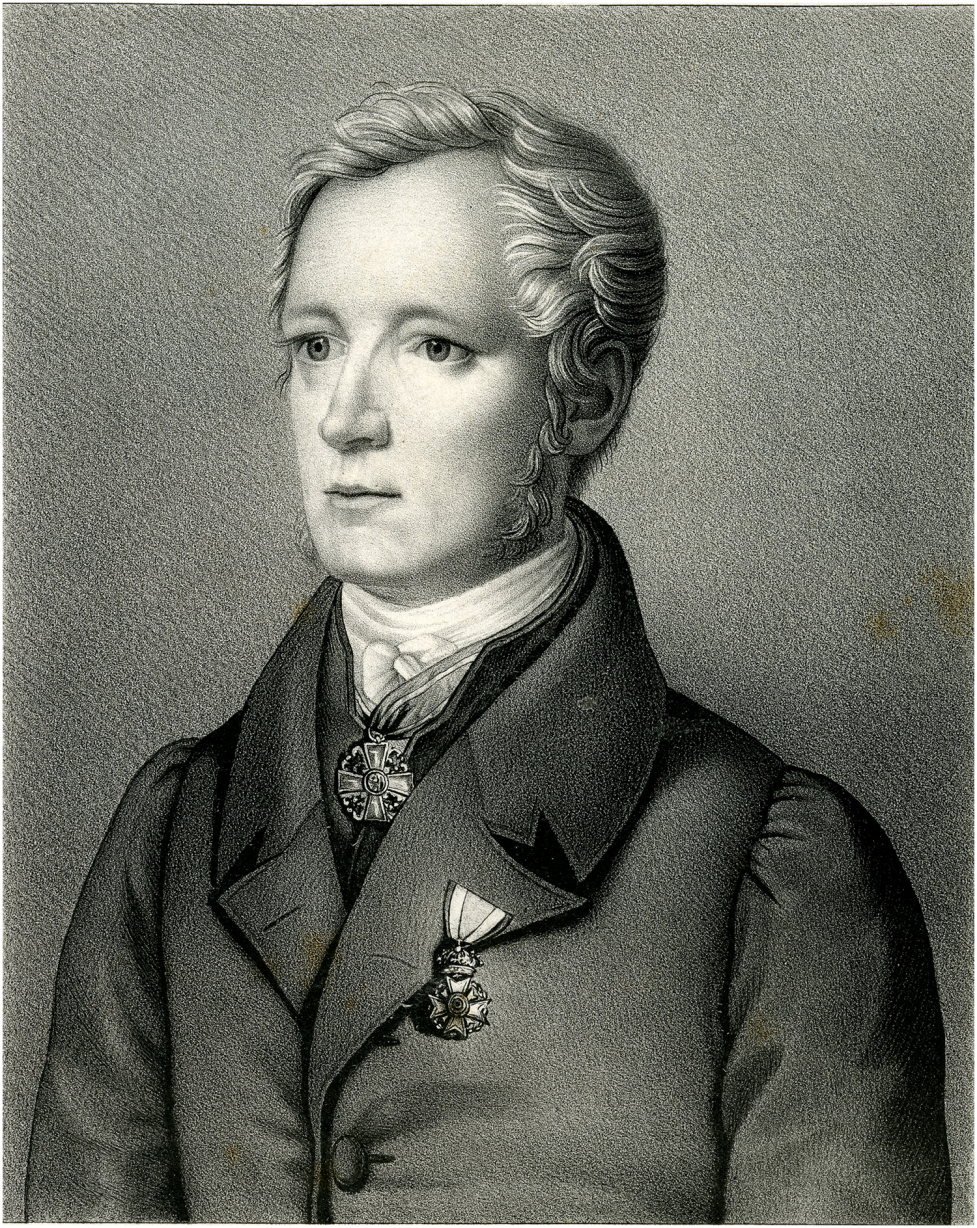
Friedrich Tiedemann (1781–1861). Image available from: http://collections.countway.harvard.edu/onview/files/original/f728617be29249bdbb122977dc683740.jpg, courtesy of the Center for the History of Medicine at the Countway Library, Harvard University.

Tiedemann’s fully published data (S1 Data part 4 and part 5) produce a remarkably similar racial ranking of cranial capacity as the one Morton presented in *Crania Americana*, with Caucasians at the top and Africans (“Aethiopians”) at or near the bottom, if one calculates the average for each racial group (Tables 2 and 3). Even though he favorably discussed previous anatomical investigations that compared, for example, the average weight of the male with the female brain [76,77], Tiedemann never calculated average differences of brain weight among racial groups (others would do so with his data later, both in criticism [80] and support [24] of his research.) And just as Tiedemann overlooked the average, Morton overlooked the ranges in organizing his findings [60,61,63]. Although both diligently explained their methods of measurement, neither Tiedemann nor Morton justified their respective choices of statistics upon which to base their differing interpretations, whether ranges or averages [11]. Their tacit, perhaps unconscious, assertions about the explanatory validity of different statistics of variation provided each a scaffold for their respective, profoundly opposed, conclusions.

**Table 2:** Descriptive statistics of Tiedemann’s brain weight data, from Tiedemann (1836). Race groups and the ordering of their presentation follow Tiedemann (1836), format of data presentation follows Morton (1839); see Table 1. See S1 Data (part 4) for all of Tiedemann’s (1836) data.

	Number of Skulls	Mean	Largest	Smallest
**Aethiopian**	41	37.17	54.32	24.96
**Caucasian**	117	39.59	57.49	27.81
**Mongolian**	20	38.41	49.17	25.04
**American**	27	39.15	59	31.84
**Malay**	43	38.56	49.22	19.35

“Number of Skulls” is the number of skulls measured by Tiedemann for this race group. “Mean” is the mean weight of millet seed fit into brain case for this race group, measured in “apothecary weight” ounces in which 1 pound equals 12 ounces, 1 ounce equals 8 drachms, and 1 drachm equals 60 grains, following Tiedeman (1836), page 500 [76]. “Largest” is the largest weight for this race group. “Smallest” is the smallest weight for this race group. Note that Tiedemann’s measures were calculated by weight, whereas Morton’s were calculated by volume. Tiedemann filled the brain case with millet seed and weighed the amount of millet seed required to fill the skull. Irrespective of methodological differences, that Tiedemann’s and Morton’s relative ranking of the racial means match is remarkable given their different interpretations of their data. Unlike Morton, Tiedemann never published racially grouped means for his data.

**Table 3:** Descriptive statistics of Tiedemann’s brain weight data, from Tiedemann (1837). For definitions, see Table 2. These data from Tiedemann’s expanded (1837) publication on brain weight across the races show that an increased sample size resulted in some differences in the ranking of mean cranial size: Tiedemann’s 1836 data (from 248 crania) show a ranking, from largest to smallest, of Caucasian > American > Malay > Mongolian > Aethiopian, while Tiedemann’s 1837 data (from 489 crania) show a ranking of Caucasian > American > Mongolian > Aethiopian > Malay. As in 1836, Tiedemann (1837) did not publish racially grouped means.

	Number of Skulls	Mean	Largest	Smallest
**Aethiopian**	88	37.59	54.32	24.96
**Caucasian**	208	39.48	57.49	27.81
**Mongolian**	49	38.11	49.17	13.68
**American**	35	39.02	59	26.22
**Malay**	109	37.53	49.22	19.31

At the end of long tables detailing skull measurements, Tiedemann execrated the slave trade as “the chain which bound Africa to the dust,” commended Great Britain’s act of abolition in 1833, and lauded the self-government of “free Negroes” in Africa and the Caribbean [76]. Without comment on present political concerns, Morton concluded *Crania Aegyptiaca* (1844) with an argument that racial differences, including those of cranial size, could not change through time. On the final page, Morton elliptically adds that the Negro’s “social position in ancient times was the same that it now is, that of servants and slaves” [61]. The “American School of Ethnology,” which claimed Morton as its founder after his death in 1851 [81,82,83], became the most virulent organ of scientific racism through the American Civil War and left a lasting imprint in later American and European racist thought [6,84]. In contrast, Anténor Firmin (1850–1911), perhaps the first black anthropologist, wrote that “the future will bring increasingly convincing proof that Tiedemann was right” [85].

## Conclusion

Gould’s major argument about Morton’s biased data is rendered highly problematic by the seed data presented here, but, even so, Morton’s work cannot be regarded as unbiased science (S6 Text). Morton’s conclusions cannot be extricated from his biases, no matter the fault in his seeds. Morton, Tiedemann, and other 19th century investigators sought to empirically ground ethical and political questions about the meaning of human difference in the measurement of skulls. The divergence of Morton’s and Tiedemann’s conclusions despite the similarity of their results shows not only that cranial race science could accommodate diametrically opposed interpretations, both for and against racial equality [86,87,88,89] but that those interpretations were underdetermined by the data. Whether as a tool to bulwark or to undermine hierarchy and oppression, this science was inevitably bound up with questions about slavery, colonialism, and differential human worth. Not inevitable, however, were the answers to these questions read from careful records of seed and lead shot packed into and poured out of a few hundred skulls.

Attention to the history and broader social context of race science reveals that such measurements are never innocent of an armature of assertions and assumptions that give them meaning, making bias much more than just a potential property of data [57]. As science is a historically, culturally, and socially situated endeavor [90,91], bias is an abiding factor in framing inquiry, forming concepts, generating questions, and designing and implementing methods, as well as interpreting results. Countervailing these forces requires “vigilance and scrutiny” [36], as Gould suggested, and a critical and diverse community of investigators [92,93] in which the open presentation of data and procedures is a norm [36,58]. As for Dr. Samuel George Morton, the accuracy of his cranial measurements neither explains nor excuses the racism constitutive of his thought and its legacy, cautioning us to remember that “unbiased data” cannot be equated with unbiased science.

## Supporting information

S1 Text Race terms. (DOCX)

S2 Text Gould reconstruction error. (DOCX)

S3 Text Gould seed–shot error. (DOCX)

S4 Text Notes on Morton’s handwritten IC. IC, internal capacity.
(DOCX)

S5 Text Additional information on seed error. (DOCX)

S6 Text Gould’s bias. (DOCX)

S1 Data 1. Morton handwritten IC data; 2. Morton new seed–shot comparisons; 3. ANOVA results; 4. Tiedemann’s 1836 cranial data; 5. Tiedemann’s 1837 cranial data. IC, internal capacity.
(XLSX)

## Abbreviation

IC: internal capacity
