# Supplementary material for: The fault in his seeds: Lost notes to the case of bias in Samuel George Morton’s cranial race science
Source: PLoS Biol. 2018 Oct 4;16(10):e2007008. doi: 10.1371/journal.pbio.2007008 (PMC6171794; doi:10.1371/journal.pbio.2007008)
Supplement: S1 Text — (DOCX) [file pbio.2007008.s001.docx]

Many historical terms used to classify racial differences are understood today to be not only offensive, but also arbitrary and incorrect. For the sake of consistency and clarity, this paper uses “African” to refer to the group that Morton labeled “Ethiopian” in 1839 [1]. Later, Morton [2, p. v-vii] renamed this group the “Negro” group, which nominally recognized the non-Africans in this group, including, for example, Native Australians, even though multiple non-African populations were part of Morton’s 1839 definition of the “Ethiopian” racial group [1, p. 86]. However, only African crania are relevant to comparison between seed and shot measures, as only sub-Saharan African crania were measured in the “Ethiopian” mean in 1839 [1, pp. 260-261].

**References**

[1] Morton SG. Crania Americana; or, A Comparative View of the Skulls of Various Aboriginal Nations of North and South America: to Which is Prefixed an Essay on the Varieties of the Human Species. Philadelphia: J. Dobson; 1839.

[2] Morton SG. Catalogue of Skulls of Man and the Inferior Animals, Third Edition. Philadelphia: Merrihew and Thomson Printers; 1849.
