# Supplementary material for: The fault in his seeds: Lost notes to the case of bias in Samuel George Morton’s cranial race science
Source: PLoS Biol. 2018 Oct 4;16(10):e2007008. doi: 10.1371/journal.pbio.2007008 (PMC6171794; doi:10.1371/journal.pbio.2007008)
Supplement: S2 Text — (DOCX) [file pbio.2007008.s002.docx]

Gould’s notes for his 1978 *Science* article on Morton [1], in the Stanford University Libraries Department of Special Collections & University Archives (Stephen Jay Gould Papers, 1899-2004, Coll. M1437, Box 354, Folder 5, “*Mismeasure of Man*, Morton,” finding guide: http://pdf.oac.cdlib.org/pdf/stanford/mss/m1437.pdf, examined by author in February, 2014) document Gould’s sample of 18 Africans and confirm that Lewis et al.’s [2] *re-reconstruction* of Gould’s reconstruction (which Gould does not detail in publication) is accurate. However, Gould’s reconstruction is almost certainly incorrect, based on Morton’s stated criteria of inclusion into the 1839 sample [3]. Morton certainly did not include, as Gould assumes, an “Oceanic Negro” (#435) or “Mixed Race Sambo” from Venezuela (#81) in his *Crania Americana* Ethiopian mean. First, Morton wrote in *Crania Americana* that “The Ethiopians were all unmixed Negroes...”, [3, p. 261] thus the “Mixed Race Sambo” should be excluded. Second, pages 86 – 88, 91 – 93, and 260-261 of *Crania Americana* show that Morton did not measure “Oceanic Negroes” as a part of his “Ethiopian” mean. Thus, this individual should be discharged as well. Moreover, Morton excluded both these individuals from his 1849 summary tables on “Negroes” [4]. Thus, consistency with Morton’s categories suggests that the number of re-measured African crania is at most 16, not 18 as suggested by Gould.

**S2 Table 1: Gould’s Reconstruction of Morton’s Sample of Re-measured African Crania**

See Lewis et al. [2, S3 Data: 1. African Seed Shot], from which this table is copied) and see key below. Note that individuals #81 and #435 do not meet Morton’s criteria for measurement as “Ethiopians” (ie. Africans).

| **Specimen #** | **Affiliation** | **IC** | **Notes** |
| --- | --- | --- | --- |
| 1 | Negro-US | 83 |  |
| 2 | Negro-US | 83 |  |
| 55 | Negro Lunatic | 89 |  |
| 63 | Negro Lunatic | 84 |  |
| 69 | Negress | 80 |  |
| 74 | Negro | 76 |  |
| 548 | Negro - St. Domingo | 86 |  |
| 549 | Negress | 83 |  |
| 421 | Native African | 88 |  |
| 422 | Native African | 80 |  |
| 423 | Native African | 85 |  |
| 580 | Native African | 67 | boy of 16 (Morton 1849 includes this specimen in his Native African summary mean) |
| 645 | Native African | 97 |  |
| 646 | Native African | 77 |  |
| 647 | Native African | 98 |  |
| 648 | Native African | 88 |  |
| 435 | Oceanic Negro India | 77 |  |
| 636 | Mixed Race/Indian/Negro | 81 | "Sambo" obtained from Venezuela |
|  | ***MEAN*** | ***83.44*** |  |

Gould’s reconstruction of the Caucasian sample is also questionable: Gould’s notes show that he included a “Cholo or Hispano-Peruvian” (# 61) in his Caucasian sample. This inclusion is curious, since Morton specifically lists this specimen as a “Mixed Race” individual [4, p. vi], not a Caucasian. Lewis et al. [2, S3 Data, 2. Euro Seed Shot] reconstruct Gould’s sample of 19 Caucasian crania, but identify more Caucasian crania in the *Catalogue* from the *Crania Americana* period than Gould does. Gould neglects to include an Englishman (# 539) or Celtic-Irish (# 42). Lewis et al. (2011) reproduce Gould’s mean, but by including those 2 individuals and not including the “Cholo or Hispano-Peruvian” (#61) and a “German?” (#706), which Gould’s notes reveal that he did, in fact, include. Gould’s exclusion of #42 and #539 and his erroneous inclusion of #61 inflates the likely re-measured sample of Caucasians from 19 to 20 crania.

**S2 Table 2: Gould’s Reconstruction of Morton’s Sample of Re-measured Caucasian Crania**

See Lewis et al. [2, S3 Data, 2. Euro Seed Shot] and see key below. Note that specimen #61 does not meet Morton’s criteria for measurement as a Caucasian, and #42 and #539 do, but these were excluded by Gould.

| **Specimen #** | **Affiliation** | **IC** | **Notes** |
| --- | --- | --- | --- |
| 7 | Anglo-American | 83 |  |
| 14 | Anglo-American | 85 | lunatic |
| 18 | Celtic Irish | 78 |  |
| 21 | Celt | 93 |  |
| 23 | Guanche | 85 |  |
| 24 | Anglo-American | 82 |  |
| 37 | German | 90 |  |
| 45 | Anglo-American | 91 | lunatic |
| 52 | Celtic Irish | 82 |  |
| 57 | Celtic Irish | 82 | lunatic |
| 58 | German | 87 | lunatic |
| 59 | Anglo-Saxon | 99 |  |
| 61 | Cholo or Hispano-Peruvian | 95 |  |
| 62 | Englishman | 92 | lunatic |
| 80 | Englishman | 91 |  |
| 434 | Dutchman | 114 |  |
| 552 | Anglo-American | 97 |  |
| 706 | German? | 94 |  |
| 724 | White woman | 81 | unknown history |
|  | ***MEAN*** | ***89.53*** |  |

**Key**

Data is from Morton (1849) [4]

IC = Cranial capacity, in cubic inches, measured with shot

Note that Morton (1849) [4] specifically included “lunatics” in his cranial capacity means, while excluding “idiots”

**References**

[1] Gould SJ. Morton's ranking of races by cranial capacity: Unconscious manipulation of data may be a scientific norm. Science. 1978;200: 503-509.

[2] Lewis JE, DeGusta D, Meyer MR, Monge JM, Mann AE, Holloway RL. The Mismeasure of Science: Stephen Jay Gould versus Samuel George Morton on Skulls and Bias. PLoS Biol. 2011;9(6): e1001071.

[3] Morton SG. Crania Americana; or, A Comparative View of the Skulls of Various Aboriginal Nations of North and South America: to Which is Prefixed an Essay on the Varieties of the Human Species. Philadelphia: J. Dobson; 1839.

[4] Morton SG. Catalogue of Skulls of Man and the Inferior Animals, Third Edition. Philadelphia: Merrihew and Thomson Printers; 1849.
