# Supplementary material for: The fault in his seeds: Lost notes to the case of bias in Samuel George Morton’s cranial race science
Source: PLoS Biol. 2018 Oct 4;16(10):e2007008. doi: 10.1371/journal.pbio.2007008 (PMC6171794; doi:10.1371/journal.pbio.2007008)
Supplement: S3 Text — (DOCX) [file pbio.2007008.s003.docx]

Gould’s supposed demonstration of the implausibility of the African seed-to-shot correction being due to sample differences [1, p. 507] does not account for one of its necessary assumptions: since *the remaining skulls in Morton’s 1839 sample did not come from Morton’s collection*, one must assume the similarity of this outstanding sample of skulls to the ones later accessioned by Morton for Gould’s comparison to hold. Gould should have instead asked (but could not have answered with the information he presented) *whether the crania that Morton borrowed* yield a mean with shot that is sufficiently small to account for the difference of seed and shot I.C. for African crania. Kaplan et al. [2, p. 4], in their insightful article, apparently entrench this mistake with their procedure of randomly sampling Morton’s *Catalogue* (1849) [3] to evidence the “heroic assumptions” necessary to account for the African seed-to-shot correction without bias. This exercise fails to recognize that *the skulls in question were never in Morton’s collection*. Irrespective of whether the number of “missing” Africans is 13 or 11 (S2 Text), the possibility that such a small number of human crania could possess a significant sampling bias in relation to the actual diversity present in whatever arbitrary “population” Morton equated with his racial categories of “Ethiopian” [4] or “Negro” [3] (S1 Text) is quite high, especially given the opportunistic nature of Morton’s collecting practices.

**References**

[1] Gould SJ (1978) Morton's ranking of races by cranial capacity: Unconscious manipulation of data may be a scientific norm. Science. 1978;200: 503-509.

[2] Kaplan JM, Pigliucci M, Banta JA. Gould on Morton, Redux: What can the debate reveal about the limits of data? Stud Hist Philos Biol Biomed Sci. 2015;52: 22–31.

[3] Morton SG. Catalogue of Skulls of Man and the Inferior Animals, Third Edition. Philadelphia: Merrihew and Thomson Printers; 1849.

[4] Morton SG. Crania Americana; or, A Comparative View of the Skulls of Various Aboriginal Nations of North and South America: to Which is Prefixed an Essay on the Varieties of the Human Species. Philadelphia: J. Dobson; 1839.
