# Supplementary material for: The fault in his seeds: Lost notes to the case of bias in Samuel George Morton’s cranial race science
Source: PLoS Biol. 2018 Oct 4;16(10):e2007008. doi: 10.1371/journal.pbio.2007008 (PMC6171794; doi:10.1371/journal.pbio.2007008)
Supplement: S4 Text — IC, internal capacity. (DOCX) [file pbio.2007008.s004.docx]

Morton’s handwritten notes in his personal copy of his 1840 *Catalogue* appear to record seed I.C. measurements taken before spring 1841 [1]. One piece of evidence for this claim is that Morton’s handwritten I.C. measurements, which are written next to the printed entry for the corresponding cranium, almost all differ from the later published shot I.C. measurements for the same cranium [2]. However, in 2 (out of 51 total) cases the handwritten I.C.s written next to the printed entries for the crania in Morton’s personal *Catalogue* (1840) [3] match their later published shot measurements. These two cases are #848 and #860, both of which are Ancient Egyptians. Although it is possible that a seed measurement could match the later shot measurement, these crania may have been measured in shot rather than seed, along with #847 and #862, which are also both Ancient Egyptians. The latter two only differ slightly from later published shot measures (S1 Data: 1. Morton Handwritten IC). These four crania were *not* included in *Crania Americana* [4]. Although the I.C. for these crania are handwritten in ink in Morton’s personal *Catalogue* like the seed measurements, Morton measured crania published in *Crania Aegpytiaca* (1844) [5] with shot and perhaps inscribed these shot I.C.s in his *Catalogue* before deciding to write them elsewhere, as suggested by Morton’s having visibly crossed out the places in which to write the I.C. for many Egyptian crania, the entries for which are printed in his personal *Catalogue* (eg. see handwritten notes in Morton’s 1840 Catalogue [3, p. 24]). Plausibly, once Morton realized that the shot measures were different than the seed measures, he did not want to confuse them by writing shot measures in the same document in which seed measures were already written.

In addition to the likely inclusion of four shot measurements next to the printed entries for crania in Morton’s personal 1840 Catalogue, there are also a series of handwritten (in pencil) I.C.s on the inside of the cover and first page of the book (listed as “Front Cover” in S1 Data: 1. Morton Handwritten IC). Many of these penciled I.C.s on the front cover correspond to crania published in *Crania Aegyptaica* but not *Crania Americana*. The penciled I.C.s match later published lead shot measurements for the same crania quite well, suggesting that these marks show Morton’s initial comparison of lead shot and seed I.C.s. Comparing the 19 (out of 20 total) legible pencil I.C. measures to later published I.C. shot measures shows that they differ on average by only -0.39 in^3^ from published shot measures for the same crania, and never more than -3 in^3^, which is a plausible range of errors with the same shot measurement method, as suggested by the experiments of Michael [6] and Lewis et al. [7]. Interestingly, two cases, those of #37 and #42, have both a handwritten I.C. entry in the pages of the 1840 *Catalogue* and a different handwritten pencil I.C. inscribed on the cover (S1 Data: 1. Morton Handwritten IC). The pencil I.C. matches later published lead shot measurements for the same crania, further suggesting that these penciled I.C. on the front cover of Morton’s personal 1840 *Catalogue* show Morton’s initial comparison of lead and seed I.C.

**References**

[1] Stated Meeting, April 6. Proceedings of the Academy of Natural Sciences of Philadelphia. 1841;1(1): 6-8.

[2] Morton SG. Catalogue of the Skulls of Man and the Inferior Animals in the Collection of Samuel George Morton. 3rd ed. Philadelphia: Merrihew and Thompson; 1849.

[3] Morton SG. Catalogue of the Skulls of Man and the Inferior Animals in the Collection of Samuel George Morton. 1st ed. Philadelphia: Turner and Fisher; 1840. Available from: https://biodiversitylibrary.org/page/55955799

[4] Morton SG. Crania Americana: or a Comparative View of the Skulls of the Various Aboriginal Nations of North and South America. Philadelphia: J. Dobson; 1839.

[5] Morton SG. Crania Aegyptiaca; or Observations on Egyptian Ethnography Derived from Anatomy, History and the Monuments. Philadelphia: John Pennington; 1844.

[6] Michael JS. (1988) A New Look at Morton’s Craniological Research. Curr Anthropol. 1988;29: 349–354.

[7] Lewis JE, DeGusta D, Meyer MR, Monge JM, Mann AE, Holloway RL. The Mismeasure of Science: Stephen Jay Gould versus Samuel George Morton on Skulls and Bias. PLoS Biol. 2011;9(6): e1001071.
