# Supplementary material for: The fault in his seeds: Lost notes to the case of bias in Samuel George Morton’s cranial race science
Source: PLoS Biol. 2018 Oct 4;16(10):e2007008. doi: 10.1371/journal.pbio.2007008 (PMC6171794; doi:10.1371/journal.pbio.2007008)
Supplement: S5 Text — (DOCX) [file pbio.2007008.s005.docx]

The range of variation in the total seed-to-shot corrections is even higher than Lewis et al. [1] report: one cranium, #744, shows a +17 in³ difference between the 1839 and 1849 measurement. This cranium has not thus far been recognized as having been re-measured between 1839 and 1849, drawing attention to the necessity of close attention to Morton’s records and collecting practices in reconstructing his measurements.

Among the crania that he lent to Morton, Combe describes the “Black-Foot Indians”:

“Black-Foot Indians, - Mr. Catlin has kindly presented me with two skulls of Black-Foot Indians, from the base of the Rocky Mountains, sent to him by Mr. Mackenzie. They approach pretty closely to the Caucasian variety in form, but they are smaller than the skulls of the Anglo-Saxon race. … Dr. Morton has not seen any of the skulls of this tribe, and I have lent them to him to be drawn for his work” (Combe in Morton 1839 [2, pp. 222-223]).

These crania escaped inclusion in the mean of re-measured Americans reported by Lewis et al. [1] because although Morton measured them in 1839, he only accessioned and assigned them “specimen numbers” later. On pages 201-202 of *Crania Americana*, Morton describes these crania. Morton, eager to possess “the only two heads [he had] ever seen of [that] isolated nation,” [2, p. 201] accessioned them following the publication of *Crania Americana*. Before their accession, he included their I.C. (and other) measurements in *Crania Americana*. The *Catalogue* numbers of these specimens are not listed in *Crania Americana*, instead listed as “C” [2, pp. 257-259]. Two “Cotonay or Black-foot” crania appear in the *Catalogue* shortly following the entry of highest specimen number mentioned in *Crania Americana*, #723. These crania, assigned numbers #744 and #745, remain in the Morton collection. Morton provided a lithographic plate, number 40, of #744, and a description of this skull as having “received a mortal blow on the top of the head, near the junction of the parietal bones, which has penetrated into the cavity of the cranium” [2, pp. 201-202).

The author’s observation of individual #744 in the Morton collection confirms its identity as the “C” skull depicted in the plate, which is corroborated by the text of the 1849 *Catalogue* entry for this individual. The blow to the parietal bone is present, as depicted on *Crania Americana* plate 40. Interestingly, this cranium was assigned a cranial capacity of 77 in³, but the 1849 *Catalogue* [4] entry for #744 shows that it rose to 94 in³. This increase of +17in³ is five cubic inches larger than any other increase recorded among American crania between individuals measured with seed and later with shot. (The new seed-shot comparisons presented in this article include a Malay cranium with a seed-shot comparison of +13 in³.) Although individual #744 was the only Cotonay individual that Morton borrowed from Combe that was featured on *Crania Americana*’s plates, allowing for visual comparison, it is almost certain that #745 is the other “C” cranium from *Crania Americana*. That individual was assigned an I.C. of 79.5 in³ in 1839, which was reduced to 75 in³ in 1849 (-4.5 in³). To keep the re-measured American sample consistent with the one presented by Gould [4] and Lewis et al. [1], these two crania have not been included in the sample of seed-to-shot corrections presented here. Were they included, they would have increased the American seed-shot correction from +2.23 in³ to +2.29 in³, but not change ANOVA results presented in the main text (S1 Data: 3. ANOVA). That errors in seed measurements of two individuals from the same tribe could differ so significantly (-4.5in³ to +17in³) underscores the claim that the errors in the seed I.C.s were significant, but do not suggest racial bias.

**References**

[1] Lewis JE, DeGusta D, Meyer MR, Monge JM, Mann AE, Holloway RL. The Mismeasure of Science: Stephen Jay Gould versus Samuel George Morton on Skulls and Bias. PLoS Biol. 2011;9(6): e1001071.

[2] Morton SG. Crania Americana; or, A Comparative View of the Skulls of Various Aboriginal Nations of North and South America: to Which is Prefixed an Essay on the Varieties of the Human Species. Philadelphia: J. Dobson; 1839.

[3] Morton SG. Catalogue of Skulls of Man and the Inferior Animals, Third Edition. Philadelphia: Merrihew and Thomson Printers; 1849.

[4] Gould SJ. Morton's ranking of races by cranial capacity: Unconscious manipulation of data may be a scientific norm. Science 1978;200: 503-509.
