# Supplementary material for: The fault in his seeds: Lost notes to the case of bias in Samuel George Morton’s cranial race science
Source: PLoS Biol. 2018 Oct 4;16(10):e2007008. doi: 10.1371/journal.pbio.2007008 (PMC6171794; doi:10.1371/journal.pbio.2007008)
Supplement: S6 Text — (DOCX) [file pbio.2007008.s006.docx]

Finding bias in Morton’s “cranial race science” does not mean that Gould’s research aimed at “debunking” of Morton was free of bias (or that Tiedemann was free of bias, either). Scrutiny of Gould’s work has revealed that his biases did likely influence some of his research [1,2,3,4,5]. Commendably, Gould was quite open regarding the bias of his own political views [6,7]. One minor archival observation gives a clear hint as to how Gould may have viewed his work on Morton: some notes for Gould’s 1978 *Science* paper on Morton were written on the back of flyers for the “Sociobiology Study Group of Science for the People,” reflecting Gould’s expressed interest in combatting both historical and contemporary “scientific theories of innate differences” (Stanford University Archives, Stephen Jay Gould Papers [1899-2004], Coll. M1437, Box 354, Folder 5).

**References**

[1] Michael JS. A New Look at Morton’s Craniological Research. Curr Anthropol. 1988;29: 349–354.

[2] Lewis JE, DeGusta D, Meyer MR, Monge JM, Mann AE, Holloway RL. The Mismeasure of Science: Stephen Jay Gould versus Samuel George Morton on Skulls and Bias. PLoS Biol. 2011;9(6): e1001071.

[3] Kaplan JM, Pigliucci M, Banta JA. Gould on Morton, Redux: What can the debate reveal about the limits of data? Stud Hist Philos Biol Biomed Sci. 2015;52: 22–31.

[4] Junker T. Critiques and Contentions: Blumenbach’s Racial Geometry. Isis 1998;89: 498-501.

[5] Gould SJ. Response: On Mental and Visual Geometry. Isis 1998; 89: 502-04.

[6] Perez M. Evolutionary activism: Stephen Jay Gould, the New Left and sociobiology. Endeavour 2013;37(2): 104-111.

[7] Gould SJ. The Mismeasure of Man. 2nd ed. New York: W. W. Norton and Company; 1996.
